# Microtubule associated tumor suppressor 1 deficient mice develop spontaneous heart hypertrophy and SLE-like lymphoproliferative disease

**DOI:** 10.3892/ijo.2011.1311

**Published:** 2011-12-20

**Authors:** CHRISTINA ZUERN, LASZLO KRENACS, STEPHANIE STARKE, JUTTA HEIMRICH, ALOIS PALMETSHOFER, BETTINA HOLTMANN, MICHAEL SENDTNER, TOBIAS FISCHER, JAN GALLE, CHRISTOPH WANNER, STEFAN SEIBOLD

**Affiliations:** 1 Department of Medicine, Division of Nephrology, University Clinic of Wuerzburg, Wuerzburg; 2 Department of Nephrology and Hypertension, University of Erlangen-Nuernberg, Erlangen, Germany; 3 Laboratory of Tumor Pathology and Molecular Diagnostics, Szeged, Hungary; 4 Biocenter of the University of Wuerzburg, Wuerzburg; 5 Institute for Clinical Neurobiology, Rudolf Virchow Center; 6 Institute for Clinical Neurobiology, University of Wuerzburg, Wuerzburg, Germany; 7 Department of Medicine, Division of Physiology, University of Wuerzburg, Wuerzburg, Germany

**Keywords:** MTSG1, ATIP, ATBP, systemic lupus erythematosus, carcinogenesis, AT2 receptor, lymphoma, proliferation, hypertrophy

## Abstract

The microtubule associated tumor suppressor gene 1 (MTUS1) is a recently published tumor suppressor gene, which has also been shown to act as an early component in the growth inhibitory signaling cascade of the angiotensin II type 2 receptor (AT2R). In this study we report the generation of MTUS1 knock-out (KO) mice, which develop normally but reveal higher body weights and slightly decreased blood pressure levels. Twenty-eight percent of the studied MTUS1 KO mice also developed heart hypertrophy and 12% developed nephritis, independent of blood pressure levels. Forty-three percent of the MTUS1 KO mice revealed lymphoid hyperplasia affecting spleen (20%), kidney (37%), lung (23%), lymph nodes (17%), and liver (17%) accompanied with leukocytosis, lymphocytosis, and mild anemia. One animal (3%) developed a marginal zone B-cell lymphoma affecting submandibular salivary gland and regional lymph nodes. The symptoms of all mentioned animals are consistent with a B-cell lymphoproliferative disease with features of systemic lupus erythematosus. In addition, body weight of the MTUS1 KO mice was significantly increased and isolated skin fibroblasts showed increased cell proliferation and decreased cell size, compared to wild-type (WT) fibroblasts in response to depleted FCS concentration and lack of growth factors. In conclusion we herein report the first generation of a MTUS1 KO mouse, developing spontaneous heart hypertrophy and increased cell proliferation, confirming once more the anti-proliferative effect of MTUS1, and a SLE-like lymphoproliferative disease suggesting crucial role in regulation of inflammation. These MTUS1 KO mice can therefore serve as a model for further investigations in cardiovascular disease, autoimmune disease and carcinogenesis.

## Introduction

The renin-angiotensin system (RAS) and its major effector protein Angiotensin II (Ang II) are known to play a major role in the regulation of blood pressure and cardiovascular homeostasis as well as in cell growth, inflammation and angiogenesis. These physiological actions are not only linked to the cardiovascular system but also to carcinogenesis and metastasis. Studies revealed a positive effect of candesartan, a potent Angiotensin II type 1 receptor (AT1R) antagonist, on tumor growth, angiogenesis and metastasis in experimental mouse models suggesting that blockade of AT1R could be an effective anticancer strategy (1). Furthermore, findings in WT or AT1aR KO mice support the hypothesis that the AT1R might play a role in inflammation-related tumor angiogenesis (2). While the AT1R might facilitate the steps towards tumor formation, the AT2R is known to oppose the effect of the AT1R in most physiological situations. But to date, little is known about the actions of the AT2R in carcinogenesis and metastasis. However, our group has recently described the MTUS1 gene, which has now linked the AT2R signaling cascade to carcinogenesis (3). MTUS1 has shown not only to act as a tumor suppressor gene in a wide range of cancers but also to operate as an interaction partner of the AT2R and seems to act as an early component of the AT2R signaling pathway in growth inhibition (3,4).

Investigations on MTUS1, which is located at chromosome 8p21.3–22 and ubiquitously expressed, showed that MTUS1 mRNA was down-regulated in fast proliferating pancreas carcinoma cell lines, while recombinant expression of MTUS1 reduced cellular proliferation significantly (3). Since then, the expression of MTUS1 has been shown to be down-regulated in breast cancer, colon tumors, prostate cancer cell lines, ovarian cancer and head and neck squamous cell carcinoma, implicating a role in a wide range of cancer development (5–10). As recently shown by our group, MTUS1 is down-regulated in 50% of the investigated colon cancers and MTUS1 siRNA transfection in HUVEC cells results in significant increased cell proliferation (11).

Shortly after the publication of MTUS1 protein, the identical protein was described as ATIP and ATBP (12,13). MTUS1 coding sequences are distributed across 18 exons, while alternative exon usage leads to a family of at least five proteins (14). MTUS1 isoform 4 protein is exclusively detected in brain, while the other three proteins are expressed in most tissues examined (14). The most investigated protein is the MTUS1 isoform 5 protein, which derives from a coding region of 10 exons, spanning 436 amino-acids with a calculated molecular mass of 50 kDa (3,12,13). The coild-coil region of the protein was able to bind to the C-terminal tail of the AT2R, but not to other receptors tested (12,13). Recombinant expression of the protein causes anti-proliferative effects while for this effect the expression but not ligand activation of the AT2R was required (12). Furthermore, the protein seemed to traffic the AT2R from the Golgi compartment to the cell surface, implicating that the protein is required for the cell surface expression of AT2R (13). Nevertheless, MTUS1 proteins seem to have more than one signaling mechanism, since AT2R and MTUS1 are not co-localizing in all tissues (14).

A recently published study reveals that Poly(ADP-ribose) polymerase-1 (PARP-1) can activate the transcription of the MTUS1 gene and represses the AT2R transcription (15). PARP-1 is known to be involved in diseases like hypertension and inflammation, although PARP-1 deficient mice do not develop cardiac hypertrophy (16).

Investigations of the neural differentiation after stimulation with Ang II exhibit a new signaling mechanism, connecting the stimulation of the AT2R with induced MMS2 expression, which enhances neural differentiation and brain protection via the interaction between MTUS1 and Src homology 2 domain-containing protein-tyrosine phosphatase 1 (SHP-1) (17).

Newly generated mice overexpressing MTUS1 showed attenuated superoxide anion production, activation of cell proliferation signaling cascades and expression of tumor necrosis factor-α (18). After cuff placement the neointimal formation of the femoral artery was significantly smaller in mice overexpressing MTUS1 than in WT mice, implicating an important role of MTUS1 in vascular remodeling (18).

Although all these results suggest an anti-proliferative role of MTUS1 in the renin-angiotensin system and as well in carcinogenesis, additional functions and regulatory mechanism of the MTUS1 gene and proteins remain largely unknown. We therefore generated an MTUS1 KO mouse line for further investigations on cell proliferation, tumor development and cardiovascular disease.

## Materials and methods

### Generation of MTUS1 KO mice

The local Ethics Committee approved the generation of MTUS1 KO mice. Gene trapping is a high-throughput approach used to introduce insertional mutations across the genome in mouse embryonic stem (ES) cells. Such cell lines can be used for generating reporter-tagged, loss-of-function mutations in mice. The Baygenomic Consortium generated a public library of mutated murine ES cell lines using a gene trap vector which simultaneously mutates and reports the expression of the endogenous gene at the site of insertion and provides a DNA tag for the rapid identification of the disrupted gene. A data base search revealed that the stem cell line RRA048 (Baygenomics, San Francisco, USA) contains a trapped MTUS1 gene by the insertion of a β-galactosidase gene in the intron region between exon 9 and exon 10. We determined the exact localization of the vector by sequencing a PCR fragment amplified with the forward primer 5′-CTGAAGCAACACAAAACCCTCTCTC-3′ and the reverse primer 5′-CACTCCAACCTCCGCAAACTC-3′ (annealing 66°C, 36 cycles). This stem cell line was used for the generation of MTUS1 KO mice by injection into blastocysts to generate germ line chimeras. These chimeras were mated with C57Bl/6 WT mice to generate heterozygote mice, which were then mated to receive homozygote MTUS1 KO mice. The local Ethics Committee approved the generation of MTUS1 KO mice (Regierung von Unterfranken, Permit Number 621-2531.01-70/03).

### Treatment and genotyping of MTUS1 deficient mice

All mice were kept in a room where the light was 12 h on and 12 h off and the temperature was kept at 22°C. In each generation homozygotes, heterozygotes and WT mice were counted to examine embryonal lethality. For all investigations MTUS1 KO mice were compared to WT littermates.

MTUS1 KO mice were identified by PCR using 5′-CCCTGCGTTTCCAGAGTCCT-3′ as forward primer and 5′-CACTCCAACCTCCGCAAACTC-3′ as reverse primer for detecting the gene-trap vector. The WT mice were identified by amplification with the same forward primer and 5′-GGTTTGATCCCCAACACCAC-3′ as reverse primer (annealing 60°C, 34 cycles) for detecting the native MTUS1 sequence.

### MTUS1 mRNA expression

Total RNA isolation from heart tissue of 12 weeks old WT and KO mice was performed using the RNeasy Mini kit (Qiagen, Hilden, Germany) and cDNA synthesis was performed using the First Strand cDNA Synthesis kit (Fermentas, St. Leon-Roth, Germany).

For the MTUS1 mRNA detection the primers 5′-CTGAAGC AACACAAAACCCTCTCTC-3′ (forward) and 5′-TGTCTG ATGCTGCTGGTTTAGTTTC-3′ (reverse) were used. β-actin forward primer 5′-TCTACAATGAGCTGCGTGTG-3′ and reverse primer 5-′TACATGGCTGGGGTGTTGAA-3′ (annealing 60°C, 40 cycles) were used for the amplification of the β-actin housekeeping gene.

### Western blot analysis

Proteins were isolated from heart tissue of 12 weeks old WT and KO mice. Therefore, tissues were homogenized and directly lysed in 50 mM hepes pH 7.5, 50 mM NaCl, 20 mM EDTA, 1 mM MgCl_2_, 2% Triton X-100, and protease inhibitor (complete mini, Roche Diagnostics, Mannheim, Germany). Subsequently, protein concentration was determined with the Bradford reaction. Ten micrograms of protein were loaded per lane, transferred to a PVDF membrane (Amersham, Freiburg, Germany) and blocked with 5% non-fat dried milk. Primary antibodies were polyclonal rabbit anti-MTUS1 (Eurogentec, Seraign, Belgium) diluted 1:2000 and monoclonal rabbit anti-β-galactosidase (Acris, Hiddenhausen, Germany) diluted 1:2000 in TBS-T containing 5% non-fat dried milk. Secondary antibody was HRP-conjugated goat anti-rabbit (Dako, Hamburg, Germany), diluted 1:2000. For detection ECL Plus (Amersham) was used.

### X-gal staining

In KO mice, the MTUS1 gene is trapped by a β-galactosidase gene, which is now under the control of the MTUS1 promotor. Staining of β-galactosidase therefore is an easy way to analyse the expression of MTUS1 in different tissues. Liver, kidney, lung, heart, brain, spleen, adrenal gland, colon and thymus of two WT and two KO mice were isolated and tissue slices were prepared. The X-gal staining was performed as previously reported (19). The counterstaining was performed using Nuklear Fast Red (Sigma-Aldrich, Taufkirchen, Germany) and the slices were dehydrated and embedded with Histofluid (Marienfeld, Lauda, Germany).

### Long-term investigation

Six WT and six KO mice were weighed at the age of four weeks and ten months. Systolic and diastolic blood pressure was measured in six WT and six KO mice at the age of 10 months by a non-invasive tail-cuff system (Föhr, Seeheim, Germany). For each mouse five values were measured and the mean pressure was calculated. For long-term investigation, ten WT and 35 KO mice were kept until the age of 10–12 months. At this age, the organs of all animals were examined macroscopically and conspicuous organs were analysed histopathologically after paraffin embedding and H&E staining. Some sections were also silver stained for reticulin fibers using Gordon and Sweet's protocol. In addition, blood was taken and analysed of ten WT and ten KO mice and heart/body weight was calculated of six WT and six KO mice.

### Immunohistochemistry

Immunohistochemistry was performed on formalin-fixed and paraffin wax-embedded sections. For wet heat-induced antigen retrieval, dewaxed and re-hydrated sections were heated in household electric pressure cocker in 1 mmol/l Tris/10 mmol/l EDTA (pH 9.0). Following the heating, the sections were washed in Tris-buffered saline (TBS; pH 7.6) containing 10% fetal calf serum (Gibco Laboratories, Grand Island, NY) for 15 min and incubated with primary antibodies at room temperature for 75 min. Primary antibodies were polyclonal goat anti-MUM1/IRF4 (M17) and anti-Pax-5 (C-20) diluted 1:5000 (both from Santa Cruz Biotechnology, Santa Cruz, CA, USA), polyclonal rabbit anti-λ and anti-κ light chain (Dako, Glostrup, Denmark) diluted 1:10.000 in TBS containing 10% FCS and 0.1% (wt/vol) NaN_3_. Link antibody was HRP-conjugated goat anti-rabbit (Dako) diluted 1:2000, which was followed by HRP-conjugated Novolink Polymer (Leica Mycrosystems). The peroxidase reaction was developed with 3.3′ diaminobenzidine tetrahydrochloride (Sigma-Aldrich, Steinheim, Germany)/0.03% (vol/vol) H_2_O_2_ and the sections were counterstained with Gill's hematoxylin.

### Isolation and investigation of skin fibroblasts

The skin of three WT and three same aged homozygote mice was removed and transferred to sterile PBS (PAA, Linz, Austria). After cutting into small pieces, the skin was incubated with 10× Trypsin/EDTA (PAA) for 30 min and suspended in FBM medium (Cambrex, Walkersville, USA) supplemented with 10% fetal calf serum (Biochrom, Berlin, Germany), insulin, rhFGF and Gentamicin/Amphotericin B (Additives FGM2, Cambrex). Three weeks after isolation the fibroblasts were split for the first time. X-gal staining was performed as described above. For the proliferation assay 1×10^5^ cells/well of three WT and three KO cell lines were plated in a 6-well plate. After 48 h incubation the cells were collected and cell concentration was specified by cell counter. Furthermore, the size of the cells was measured by FACS analysis (Beckton-Dickinson, Heidelberg, Germany).

For investigation of the proliferation rate of WT and KO cells in response to various growth factors, 1×10^3^ cells were seeded in a 96-well plate in FBM medium with all supplements. After 48 h the medium was removed and the cells were treated with either FBM medium with all supplements, FBM medium with 1% FCS, FBM medium with 1% FCS and 0.1% rhFGF or FBM medium with 1% FCS and 0.1% insulin. Cell proliferation was determined using the Cell Proliferation ELISA (Roche, Mannheim, Germany) according to the manufacturer's manual after 24 h of incubation.

### Statistical analyses

Data are presented as means ± SEM and analyzed by Student' t-test. A P<0.05 was considered significant.

## Results

### Generation and characterization of MTUS1 KO mice

**Figure 1:**
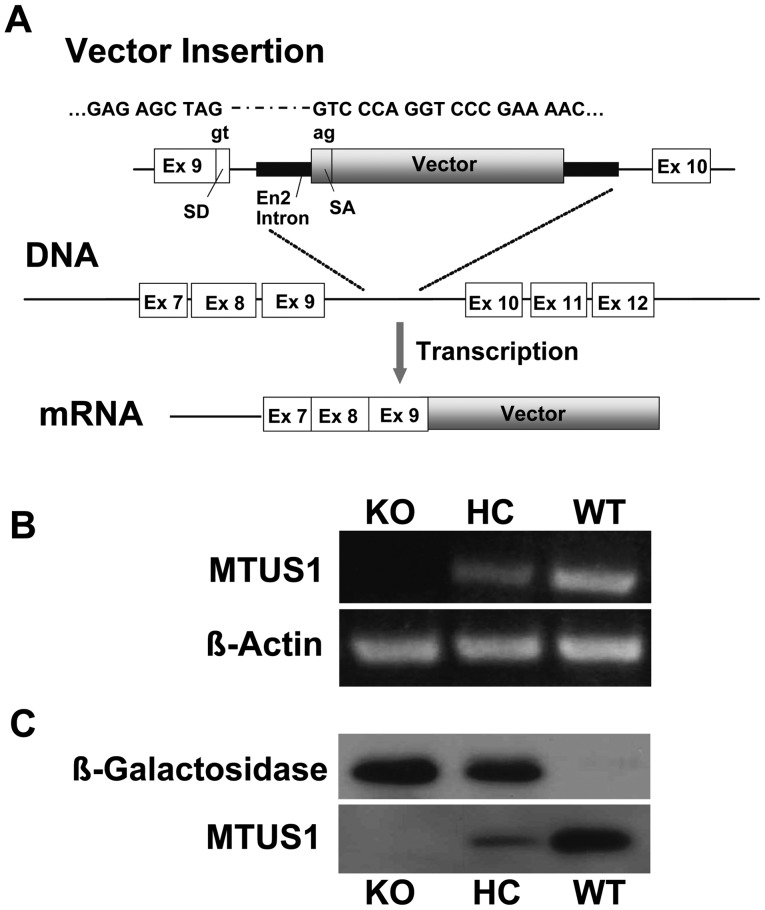
(A) The gene trap vector was inserted into the intron sequence between exon 9 and 10. (B) RT-PCR analysis of mRNA isolated from heart tissue revealed high expression of MTUS1 in wild-type (WT) mice, reduced expression in heterozygote (HC) mice and no expression in homozygote (KO) mice, while β-actin levels demonstrated equal mRNA amount. (C) Knockout of MTUS1 protein expression was confirmed by Western blot analysis in heart tissue of homozygote (KO) and heterozygote (HC) mice compared to wild-type (WT) mice. MTUS1 KO mice showed β-galactosidase protein expression instead of MTUS1 expression and HC mice revealed reduced MTUS1 expression and β-galactosidase expression.

For characterization of the generated KO mice, we first investigated the correct insertion of the β-galactosidase vector into the MTUS1 gene and confirmed the functional KO on mRNA and protein level. Sequencing results confirmed the correct insertion of the β-galactosidase vector into the intron region between exon 9 and exon 10 of the MTUS1 (Fig. 1A). Since MTUS1 is known for its anti-proliferative effect, a possible embryonal lethality had to be examined. In the F1 generation of the chimeric mice 18 heterozygotes and 14 WT mice were born alive, in the F2 generation 23% WT, 58% heterozygote and 19% homozygote mice were born alive. Therefore, Mendelian law was confirmed and an obviously higher embryonal lethality of homozygote or heterozygote mice was not observed.

RT-PCR analysis of WT and KO mice showed no detectable MTUS1 expression in the MTUS1 deficient mice, while the WT mice revealed normal mRNA expression (Fig. 1B). Knockout of MTUS1 protein expression was confirmed by Western blot analysis in the heart tissue of MTUS1 KO mice compared to WT mice. MTUS1 KO mice showed β-galactosidase protein expression instead (Fig. 1C).

### Tissue distribution of the MTUS1 expression

MTUS1 is known to be expressed in various tissues at moderate or high levels. With the X-gal staining in MTUS1 knockout mice the tissue distribution could be investigated more precisely, since in MTUS1 knockout mice the β-galactosidase gene is under the control of the MTUS1 promoter.

**Figure 2:**
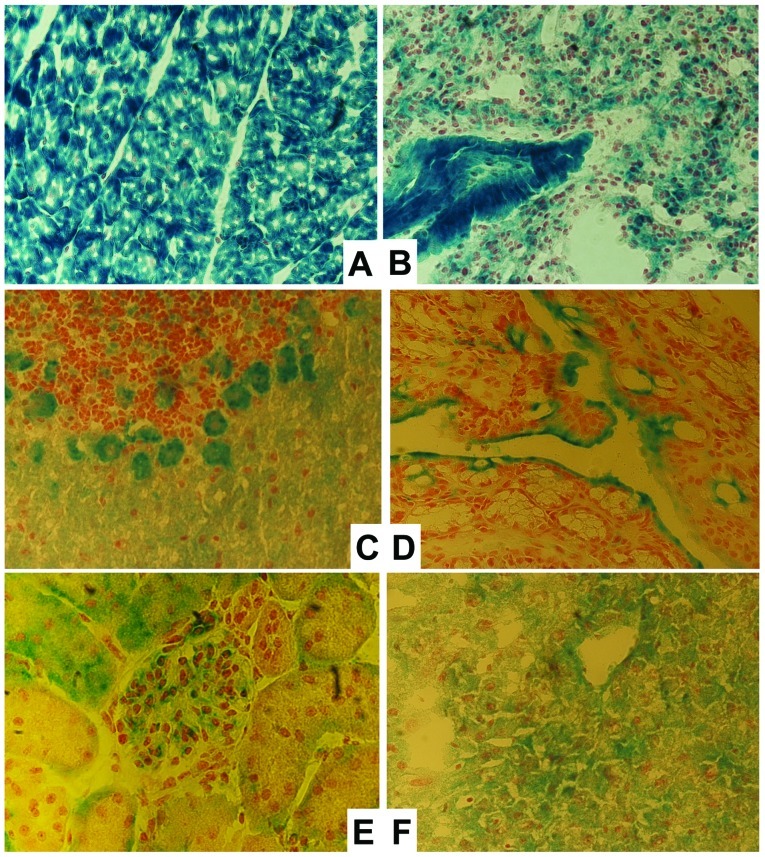
X-gal staining (blue colour) stands for β-galactosidase activity and therefore MTUS1 expression. The highest expression was determined in the heart, where all cardiomyocytes as well as the endothelial cells were stained (A). In lung and colon the highest staining was observed in the epithelial and endothelial cells (B and D). In brain the Purkinje cells showed X-gal staining (C). In the kidney the glomeruli and the proximal tubulus cells were clearly stained (E). The hepatocytes revealed a light and equal staining (F).

The highest expression was determined in the heart, where all cardiomyocytes as well as the endothelial cells were intensively stained. In lung and colon the highest staining was observed in the epithelial and endothelial cells. In the kidney especially the glomeruli and the proximal tubulus cells were clearly stained. The hepatocytes revealed a slight homogeneous staining, while in brain the Purkinje cells were clearly stained (Fig. 2). Spleen and adrenal gland were discretely stained only in the area of the capsule and some connective tissue (data not shown). The thymus had some net-like strands of epithelial cells, where staining was also obtained (data not shown). X-gal staining was performed in WT animals as negative controls as well, but as expected no staining was detected (data not shown).

### Long-term investigations

For long-term investigations, 10 WT mice and 35 KO mice were housed for 10–12 months. The behaviour of the KO mice was not altered during this observation period.

**Figure 3:**
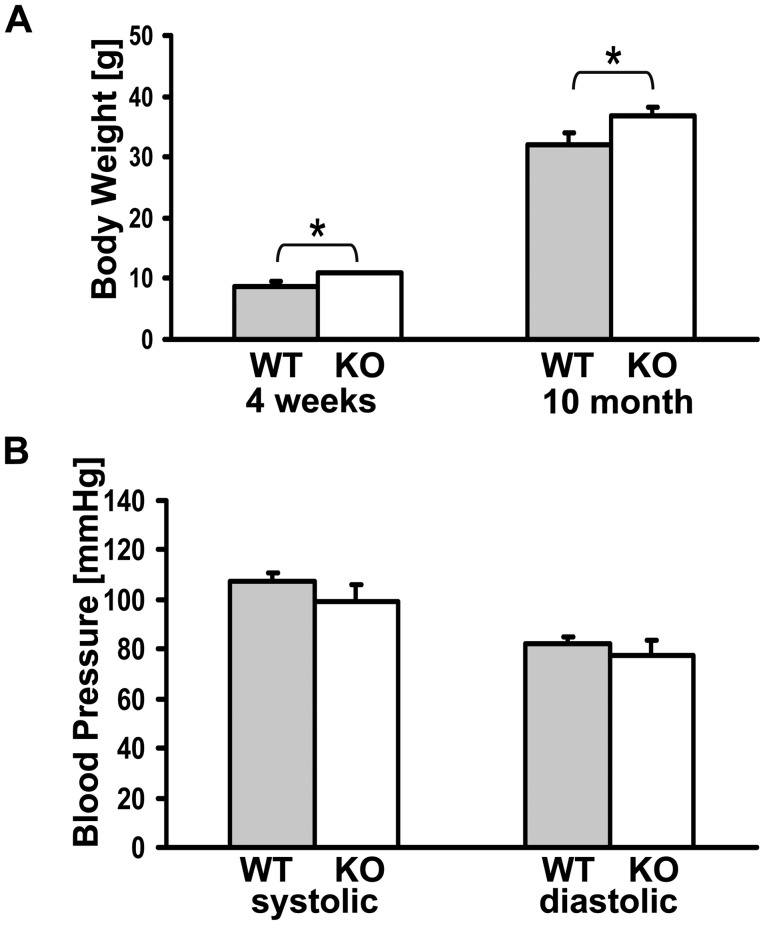
(A) At the age of four weeks and ten months the MTUS1 KO mice revealed a significantly higher body weight (p=0.028 and p=0.036). Data are means + SEM. (B) Blood pressure was analysed by a non-invasive tail-cuff system of six MTUS1 KO mice compared to WT littermates at the age of four weeks and ten months. The measurement missed the significance level (p=0.101 systolic and p=0.087 diastolic). Data are means + SEM from 5 measurements in each mouse.

For analysis of the body weight of the mice, six WT and six KO mice were measured at the age of four weeks and 10 months. Even at the age of four weeks, the KO mice revealed a significantly (p=0.028) higher body weight, the KO mice weighed 10.8±0.2 g and the WT mice 8.7±0.8 g. At the age of 10 months the effect was still maintained, the KO weighed 36.7±1.6 g and the WT mice 32±1.9 g (p=0.036) (Fig. 3A).

Systolic and diastolic blood pressure was compared in six WT and six MTUS1 KO mice at the age of 10 months. The mean systolic blood pressure was 100±6 mmHg in KO mice and 107±4 mmHg in WT mice whereas the mean diastolic blood pressure was 77±6 mmHg in KO mice and 82±3 mmHg in WT mice. Even if there was a trend towards lower blood pressure in MTUS1 KO mice, the result was not significant (p=0.101 for the systolic blood pressure and p=0.087 for the diastolic blood pressure) (Fig. 3B).

For the investigation of the laboratory blood values, blood samples of 10–12 months old MTUS1 KO mice were compared to WT littermates. Most serum levels of the investigated parameters, for example all electrolyte concentrations, did not significantly differ between the MTUS1 KO and the WT mice. The hematologic values were noticeably different, with higher leucocyte and lymphocyte counts in the MTUS1 KO mice compared to WT mice, while erythrocytes, hemoglobin, hematocrit and thrombocyte levels were reduced (Table I).

### Pathology of the MTUS1 KO mice

**Figure 4:**
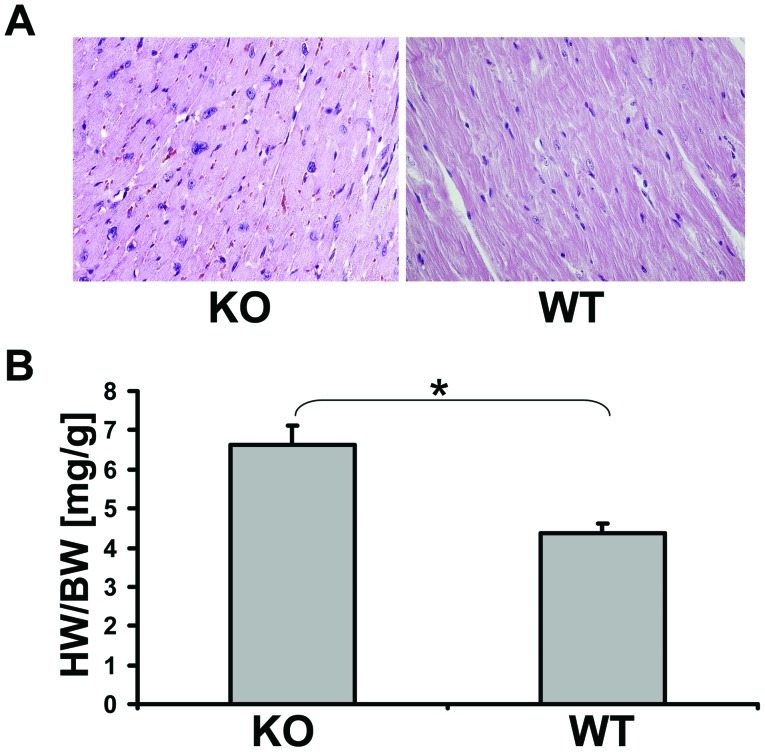
Pictures show normal WT and hypertrophic KO heart tissue with H&E staining (A, ×200). The calculation of the heart/body ratio revealed significant heart hypertrophy in MTUS1 KO mice (B, p=0.006).

**Figure 5:**
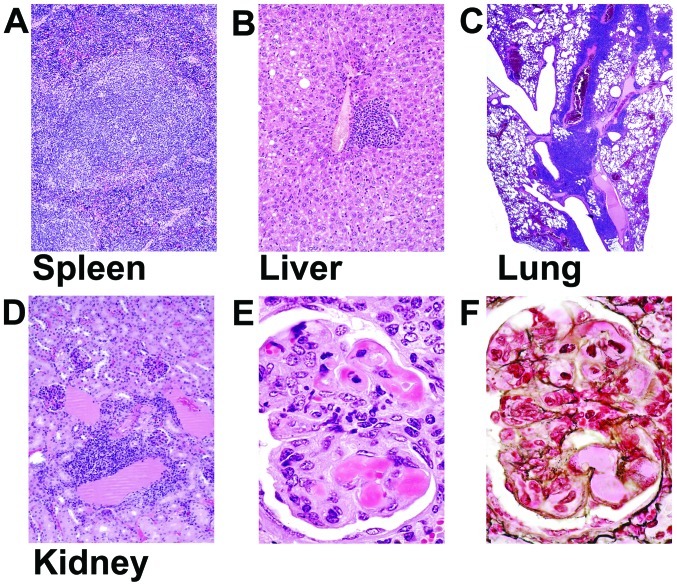
Most MTUS1 KO mice revealed lymphoid hyperplasia affecting (A) spleen (20%), (B) liver (17%), (C) lung (23%) and (D) kidney (37%). Glomerulonephritis was found in 12% of the MTUS1 KO mice. Pictures show diffuse glomerulonephritis with wire loop lesion (E) H&E staining and (F) Gordon and Sweets silver staining. (Original magnification: (A) and (D) ×100, (B) ×400, (C) ×50, (E) and (F) ×1,000).

**Figure 6:**
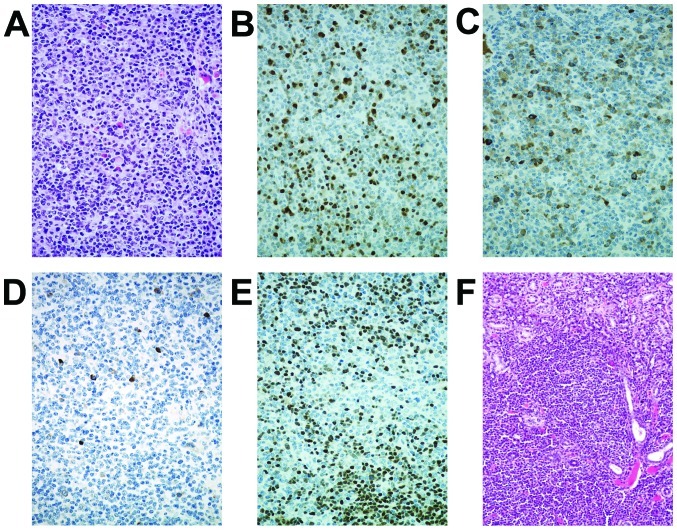
One MTUS1 KO mouse showed marginal zone B-cell lymphoma (so-called MALT lymphoma) submandibular salivary gland and regional lymph nodes. Lymphomatous lymph node infiltrate: (A) H&E staining, (B) MUM1/IRF-4, (C) κ, (D) λ, and (E) Pax5. Significant κ light chain dominance in plasmocytoid cells supports monoclonal B-cell proliferation. (F) Lymphomatous infiltrate in salivary gland is demonstrated (H&E). [Original magnification: (A-F) ×200].

None of the 10 WT mice died spontaneously during the long-term investigation of 12 months. In contrast, 37% of the KO mice died spontaneously or had to be sacrificed for ethical reasons. The autopsy of the WT mice at the age of 12 months revealed no organ anomaly and the histopathology did not demonstrate any unusual findings. Compared to WT animals, most of the KO mice revealed organ anomalies. Twenty-eight percent of the studied MTUS1 KO mice developed heart hypertrophy and 12% developed glomerulonephritis. The heart/body weight was significantly (p=0.006) increased in MTUS1 KO mice (Fig. 4). Eighty percent of the nephritic animals showed diffuse glomerulonephritis with wire-loop lesion in one and rapidly progressive glomerulonephritis with crescent formation in another animal (Fig. 5). Forty-three percent of the MTUS1 KO mice revealed multiorgan lymphoid hyperplasia affecting spleen (20%), kidney (37%), lung (23%), lymph nodes (17%), and liver (17%) accompanied with leukocytosis, lymphocytosis, and mild anemia (Fig. 5, Table I). Eight percent of the animals revealed sialadenitis and 3% insulitis. One animal (3%) developed a marginal zone B-cell lymphoma affecting submandibular salivary gland and regional lymph node (Fig. 6). The symptoms of all mentioned animals are consistent with a B-cell lymphoproliferative disease with features of systemic lupus erythematosus.

### Investigations on skin fibroblasts

**Figure 7:**
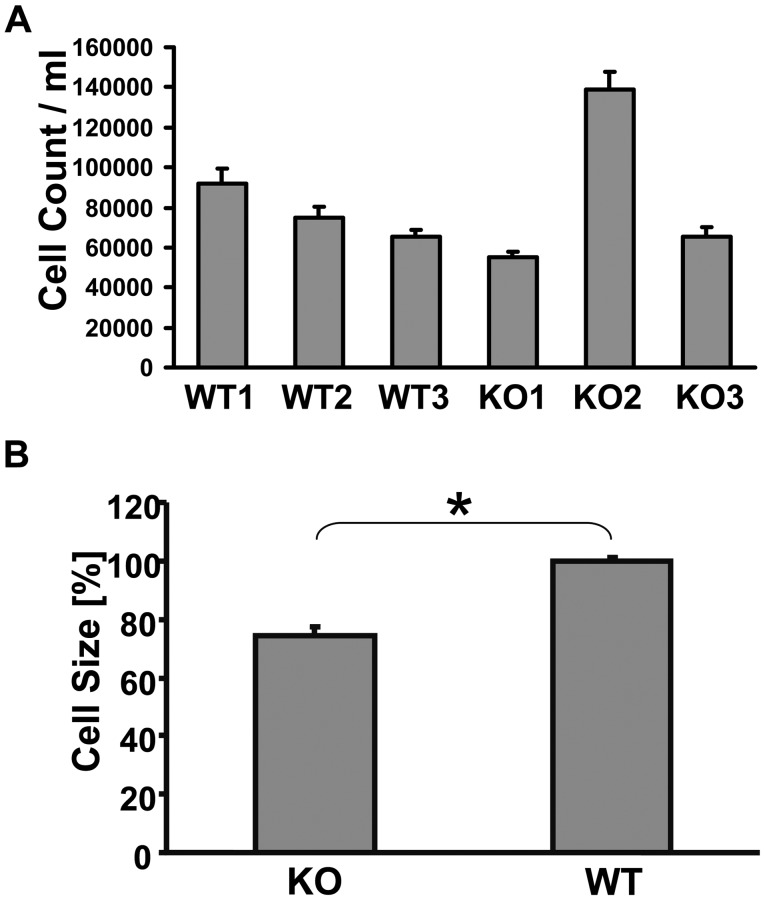
(A) Proliferation assay of skin fibroblasts of three WT mice (WT1, WT2 and WT3) and three MTUS1 KO mice (KO1, KO2 and KO3). Forty-eight hours after seeding (1×10^5^ cells/6-well), cells were harvested and counted using a Coulter Z2. Data are means + SEM from six independent experiments. (B) FACS analysis of skin fibroblasts of three WT mice and three MTUS1 KO mice. Forty-eight hours after seeding (1×10^5^ cells/6-well), cells were harvested and cells were analysed using FACS. The cell size is proportional to the forward scatter, so the data of the WT mice were equalized with 100% and the MTUS1 KO data were calculated. MTUS1 KO fibroblasts are significantly smaller than WT fibroblasts (p=0.001). Data are means + SEM from six independent experiments.

**Figure 8:**
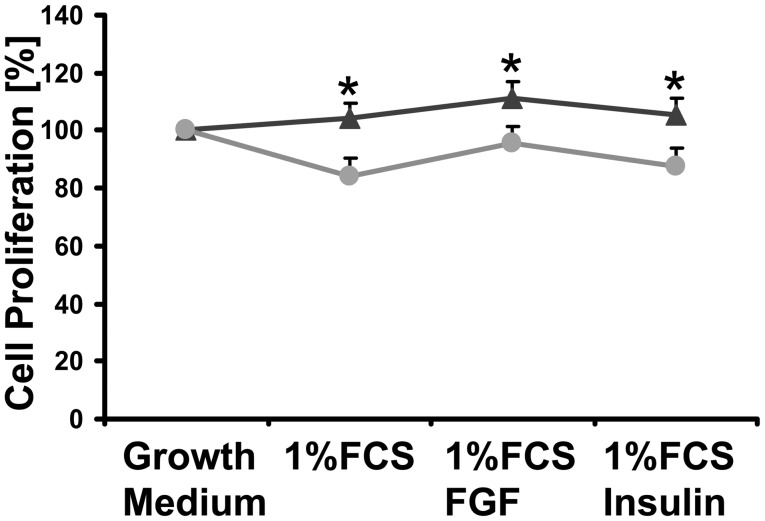
A proliferation assay of fibroblasts of three WT mice (WT1, WT2 and WT3) and three MTUS1 KO mice (KO1, KO2 and KO3) was performed. Forty-eight hours after seeding (1×10^3^ cells/96-well), cells were stimulated with FBM growth medium, FBM supplemented either with 1% FCS, 1% FCS and 0.1% rhFGF or 1% FCS and 0.1% insulin. After twenty-four hours of stimulation, cell proliferation was measured with ELISA assay. Proliferation in normal growth medium was defined as 100% in each cell line. WT fibroblasts show significant lower proliferation in 1% FCS medium (p=0.004), as well as in 1% FCS/0, 1% rhFGF (p=0.0023) and 1% FCS/0.1% insulin (p=0.011). Data are means + SEM from at least three independent experiments.

Skin fibroblasts were isolated from three WT and three MTUS1 KO mice for further *in vitro* investigation of cellular proliferation. X-gal staining was positive in fibroblasts and the MTUS1 protein seemed to be located in the cytoplasm near the nucleus. Mitotic cells were stained more intensively (data not shown). Cell proliferation of the MTUS1 KO fibroblasts were equal to the WT fibroblasts in two cell lines, but the third MTUS1 KO cell line was proliferating almost at double speed (Fig. 7A), while the cell volume of all MTUS1 KO cell lines was significant smaller (p=0.001) than the WT controls (Fig. 7B). Cell culture analyses with different growth factors were performed to further characterize the proliferation characteristics of the MTUS1 KO fibroblasts. Thereby WT fibroblasts showed much more sensitivity for FCS depletion and depletion of growth factors, resulting in a significant lower proliferation (p=0.004, p=0.0023 and p=0.011) (Fig. 8).

## Discussion

The present study reports the generation and characterization of the first mouse model deficient for MTUS1. Previously, MTUS1 has been reported to play a role in the cell proliferation as well as in cancer development (3,11). Therefore, the main purpose of the study was to prove that MTUS1 can regulate the cell proliferation and to investigate if the lack of MTUS1 alone can cause cancer development or major pathological changes.

In the long-term investigations the MTUS1 KO mice reveal two major diseases. On the one hand the KO mice develop a significant higher body weight and heart hypertrophy, furthermore, multiple damage to organs like fatty degeneration of the liver were observed. The anti-proliferative function of MTUS1, which was recently linked to its role in the AT2R pathway, may play an important role in the development of the high body weight and the hypertrophy of the heart, where X-gal staining shows the highest and abundant expression of all tested organs (12). As reported previously, the AT2R antagonizes the accelerating growth effects of the AT1R in cardiomyocytes (20). In addition, the development of heart hypertrophy in AT2 KO mice is not or only in part a consequence of hypertension, because AT2 KO mice developed hypertension, but no or only a light heart hypertrophy (21). MTUS1 KO mice show the tendency for lower blood pressure, while mice overexpressing MTUS1 show a tendency for higher blood pressure without any medical treatment (18). Both results miss the significance level, but the data are still surprising. A new signaling pathway, involving the interaction of the AT2R and PLZF protein was reported as a possible pathway in the development of heart hypertrophy (22). After binding to the AT2R in the heart, PLZF protein translocates to the nucleus and activates the transcription of p85α, the regulatory unit of P13 kinase. Growth factors such as EGF activate p85α, enhancing protein synthesis essential to cardiac hypertrophy (23). In contrast, the AT2R linked activation of SHP-1 protein down-regulates growth factors and p85α (23). A newly published study in neurons shows that MTUS1 can interact with the SHP-1 protein after AT2R binding, resulting in increased MMS2 transcription and neural differentiation (17). Previous studies show not only an interaction of MTUS1 with the AT2R but also an MTUS1 mediated inhibition of insulin, bFGF and EGF signal cascades, which lead to the activation of ERK2 (12). MTUS1 could therefore prevent heart hypertrophy in two ways. The interaction with SHP-1 could lead to a down-regulated transcription of p85α in the heart and inhibition of EGF could prevent the activation of p85α. Both issues must be addressed for further understanding the development of heart hypertrophy in MTUS1 KO mice.

One out of three MTUS1 KO fibroblast cell lines shows significantly increased cell proliferation, implicating that the increased proliferation could differ individually and might be dependent on other factors as well. Nevertheless, MTUS1 KO fibroblasts show significant higher proliferation in medium with depleted FCS concentration and lack of growth factors than control WT fibroblasts. MTUS1 is known to have anti-proliferative effects via the inhibition of insulin, epidermal growth factor and fibroblast growth factor, so the higher proliferation in MTUS1 KO cells could be due to the obviated inhibition of these growth factors and therefore a lower sensitivity to reduced growth factors in the medium (12).

The second major symptomatology in the MTUS1 KO mice revealed multiorgan lymphoid hyperplasia, splenomegaly, accompanied with glomerulonephritis, and sialadenitis in some animals. The wire loop lesion of the kidney and the histopathological investigations suggest a SLE-like systemic autoimmune disease, which was at least in one case complicated with a marginal zone B-cell lymphoma affecting submandibular salivary gland and regional lymph node. The blood values reveal higher counts of lymphocytes and leucocytes but mild anemia with enlarged erythrocytes and reduced hemoglobin levels. These results are not completely in accord with the values of a SLE disease, but the high level of lymphocytes could be due to the developement of a lymphoma. To date, nothing is known about the effects of MTUS1 in hematopoiesis or autoimmune disease like SLE, but the reported interaction with AT2R could be a hint for similar effects than the RAS pathway. Interestingly, the ACE KO mice develop mild anemia and ACE inhibitors and AT1R antagonists are sporadic reported to cause anemia and bone marrow aplasia (24). In addition, the RAS pathway is known to play a role in immune responses and inflammation. A new study in a lupus mouse line with AT1AR deficiency did not show the expected benefit in lupus nephritis, because the remaining glomerular AT1BR was stimulated and caused even more severe injury. This enhanced disease process could be prevented with losartan treatment (25). Even if randomised trials in humans are currently missing, RAS inhibition revealed a general benefit for SLE patients (26). Keeping in mind that the AT2R can antagonize the AT1R in many cases, there could be a protective effect of the AT2R and its binding proteins in SLE. MTUS1 down-regulation is now well-described in a wide range of tumor tissues, but to date there have been no studies on MTUS1 involvement in lymphoproliferative diseases like lymphoma (5–8,27). At least one animal showed SLE-like disease accompanied with MALT lymphoma, which can arise at any extranodal site, share histologic and immunophenotypic characteristics and is usually associated with chronic inflammation resulting of autoimmune disease or infection (28). In detail, 10 of 14 separate studies in human SLE patients reported a 3-to 40-fold increased risk of non-Hodgkin lymphoma and all lymphoma were of the B-cell subtype (29). In our study, the autoimmune disease could therefore be the initial step of lymphoma formation. In general, four chromosomal translocations are well known to be involved in the development of MALT lymphoma, which affect the api2, malt1 bcl10, foxp1 and IgH genes and have partly shown to cause an activation of the nuclear factor kappa B (NF-κB) pathway, suggesting a common occurance for MALT lymphomas (30). MTUS1 gene has not yet been reported to be mutated or translocated in MALT lymphoma, but its interaction partner AT2R and the RAS were recently described in various aspects of inflammation, including the NF-κB pathway (1). AT2R activation oppose the pro-inflammatory effects of the AT1R by preventing the activation of NF-κB as well as stabilizing the inhibitory protein κB through an SHP-1 dependent pathway (31,32). MTUS1 has been shown to build a complex with SHP-1 upon AT2R stimulation and to translocate into the nucleus, resulting in enhanced cell differentiation in neurons (4). In addition, NF-κB showed constitutive activation not only in SLE but also in some cancers and leukemias, substantiating the molecular link between chronic inflammation, autoimmunity and carcinogenesis (33). It is noteworthy that the microvascular permeability was decreased by activation of AT2R and increased by blockade of the AT2R (34). MTUS1 has been shown to be localized in the vascular endothelium and to interact with the AT2R to cooperate in different functions, so it would be possible that lack of MTUS1 could effect the microvascular permeability. Taking these results together, it could be hypothesised that a defiency of MTUS1 could result in an increased activation of NF-κB and increased microvascular permeability, enabling the infiltration of inflammatory or metastatic cells and therefore an increased incidence of autoimmune disease and lymphoma.

In previous studies, wide tissue distribution of MTUS1 mRNA was reported by quantitative RT-PCR and some new studies examined the down-regulation of MTUS1 in tumors such as ovarian cancer, pancreatic carcinoma or breast cancer, but to date nothing is known about the localisation of MTUS1 within these tissues (3,5,13,14,27). The X-gal staining revealed highly restrictive distribution of MTUS1 proteins. All examined tissues show a similar pattern of expression and mostly endothelial and epithelial cells were stained, whereas the stromal cells showed a much lower staining. In heart and brain the cardiomyocytes and the Purkinje cells were stained additionally. Most tumors have their origin in endothelial and epithelial cells, thus the localisation of MTUS1 could be a hint of its function as tumor suppressor gene. Expression profiles of MTUS1 proteins and the AT2R are partly overlapping for example in the endothelial cells of heart and cardiomyocytes or the epithelial cells in lung, but in colon and kidney the expression pattern seem to differ (35–38), suggesting an additional AT2R-independent pathway for MTUS1.

In conclusion, we report here the generation of the first MTUS1 KO mouse line, which develops spontaneous heart hypertrophy and SLE-like lymphoproliferative disease. In addition, skin cells of MTUS1 KO mice reveal higher cell proliferation in response to depleted FCS and growth factors. Therefore, MTUS1 KO mice further support the hypothesis of an anti-proliferative effects of MTUS1 and can serve as a model for further investigations in autoimmune disease, cardiovascular disease and carcinogenesis.

## Figures and Tables

**Table I:** Blood values of MTUS1 KO and WT mice.

	WT	KO
Sodium	150.1 (±0.74) mmol/l	148.8 (±0.99) mmol/l
Calcium	2.85 (±0.03) mmol/l	2.71 (±0.05) mmol/l
Magnesium	1.3 (±0.05) mmol/l	1.5 (±0.10) mmol/l
Inorganic phosphate	2.65 (±0.13) mmol/l	3.03 (±0.27) mmol/l
Chloride	113.1 (±0.54) mmol/l	113.3 (±1.14) mmol/l
Glucose	364.2 (±25.6) mg/dl	286.9 (±33.2) mg/dl
Urea	36.8 (±4.01) mg/dl	68.7 (±18.1) mg/dl
Uric acid	5.8 (±0.48) mg/dl	4.1 (±0.34) mg/dl
Total protein	6.0 (±0.12) g/dl	5.9 (±0.23)g/dl
Albumin	3.6 (±0.08) g/dl	3.2 (±0.14)g/dl
Cholinesterase	8033.0 (±424) U/l	8174.6 (±458) U/l
Bilirubin	0.17 (±0.03) mg/dl	0.16 (±0.02) mg/dl
Glutamate-oxalacetate-transferase (GOT)	87.7 (±7.52) U/l	158.6 (±21.6) U/l
Glutatmate-pyruvate-transaminase (GPT)	48.4 (±3.74) U/l	54.3 (±14.5) U/l
Glutamate dehydrogenase (GLDH)	9.3 (±1.52) U/l	9.1 (±2.13) U/l
Alkaline phosphatase	85.5 (±23.8) U/l	63.5 (±8.21) U/l
Lactate dehydrogenase	427.5 (±77.3) U/l	625.5 (±84.4) U/l
Creatine kinase	130.3 (±22.5) U/l	502.9 (±106) U/l
Lipase	28.8 (±3.65) U/l	20.5 (±1.80) U/l
Cholesterol	118.8 (±14.5) mg/dl	112.3 (±9.64) mg/dl
Triglyceride	107.6 (±9.75) mg/dl	97.5 (±6.40) mg/dl
HDL cholesterol	82.0 (±0.00) mg/dl	91.4 (±13.2) mg/dl
Iron	202.6 (±15.2) μg/dl	181.8 (±12.7) μg/dl
Leukocyte	5.7 (±0.64)*1000/μl	8.7 (±0.72)*1000/μl
Erythrocyte	8.9 (±0.15)*10E6/μl	7.8 (±0.32)*10E6/μl
Hemoglobin	13.5 (±0.17) g/dl	11.3 (±0.67) g/dl
Hematocrit	47.7 (±0.55)%	43.0 (±1.70)%
Mean corpusculare volume (MCV)	53.7 (±0.47) fl	55.3 (±0.68) fl
Mean corpusculare hemoglobin (MCH)	15.2 (±0.15) pg	14.3 (±0.59) pg
Mean corpusculare hemoglobin concentration (MCHC)	28.2 (±0.17) g/dl	25.9 (±1.03) g/dl
Thrombocyte	753.3 (±73.4)*1000/μl	608.4 (±53.3)*1000/μl
Lymphocyte	2.5 (±0.29)*1000/μl	3.6 (±0.29)*1000/μl

For the summary of the blood values, the samples of 10–12 months old MTUS1 KO mice were compared to WT littermates. Most serum levels of the investigated parameter, for example all salt concentrations, did not differ between the MTUS1 KO and the WT mice. The hematologic values were noticeable different. The leucocytes and lymphocytes were measured at higher levels in the MTUS1 KO mice, while erythrocytes, hemoglobin, hematocrit and thrombocyte levels were reduced (Table I).

## References

[1] Deshayes F, Nahmias C (2005). Angiotensin receptors: a new role in cancer?. Trends Endocrinol Metab.

[2] Egami K, Murohara T, Shimada T (2003). Role of host angiotensin II type 1 receptor in tumor angiogenesis and growth. J Clin Invest.

[3] Seibold S, Rudroff C, Weber M, Galle J, Wanner C, Marx M (2003). Identification of a new tumor suppressor gene located at chromosome 8p21.3-22. FASEB J.

[4] Mogi M, Iwai M, Horiuchi M (2007). Emerging concepts of regulation of angiotensin II receptors: new players and targets for traditional receptors. Arterioscler Thromb Vasc Biol.

[5] Frank B, Bermejo JL, Hemminki K (2007). Copy number variant in the candidate tumor suppressor gene MTUS1 and familial breast cancer risk. Carcinogenesis.

[6] Lee S, Bang S, Song K, Lee I (2006). Differential expression in normal-adenoma-carcinoma sequence suggests complex molecular carcinogenesis in colon. Oncol Rep.

[7] Tchatchou S, Burwinkel B (2008). Chromosome copy number variation and breast cancer risk. Cytogenet Genome Res.

[8] Ye HP, Huang N, Muzio BL (2007). Genomic assessments of the frequent loss of heterozygosity region on 8p21.3-p22 in head and neck squamous cell carcinoma. Cancer Genet Cytogenet.

[9] Simon NSL, Laurie C, Linda R (2010). Expression and function of ATIP/MTUS1 in human prostate cancer cell lines. Prostate.

[10] Rodrigues-Ferreira S, Di Tommaso A, Dimitrov A (2009). 8p22 MTUS1 gene product ATIP3 is a novel anti-mitotic protein underexpressed in invasive breast carcinoma of poor prognosis. PLoS One.

[11] Zuern C, Heimrich J, Kaufmann R (2010). Down-regulation of MTUS1 in human colon tumors. Oncol Rep.

[12] Nouet S, Amzallag N, Li JM (2004). Trans-inactivation of receptor tyrosine kinases by novel angiotensin II AT2 receptor-interacting protein, ATIP. J Biol Chem.

[13] Wruck CJ, Funke-Kaiser H, Pufe T (2005). Regulation of transport of the angiotensin AT2 receptor by a novel membrane-associated golgi protein. Arterioscler Thromb Vasc Biol.

[14] Di Benedetto M, Bièche I, Deshayes F (2006). Structural organization and expression of human MTUS1, a candidate 8p22 tumor suppressor gene encoding a family of angiotensin II AT2 receptor-interacting proteins, ATIP. Gene.

[15] Reinemund J, Seidel K, Steckelings UM (2009). Poly(ADP-ribose) polymerase-1 (PARP-1) transcriptionally regulates angiotensin AT2 receptor (AT2R) and AT2R binding protein (ATBP) genes. Biochem Pharmacol.

[16] Pillai JB, Gupta M, Rajamohan SB, Lang R, Raman J, Gupta MP (2006). Poly(ADP-ribose) polymerase-1-deficient mice are protected from angiotensin II-induced cardiac hypertrophy. Am J Physiol Heart Circ Physiol.

[17] Li JM, Mogi M, Tsukuda K (2007). Angiotensin II-induced neural differentiation via angiotensin II type 2 (AT2) receptor-MMS2 cascade involving interaction between AT2 receptor-interacting protein and Src homology 2 domain-containing protein-tyrosine phosphatase 1. Mol Endocrinol.

[18] Fujita T, Mogi M, Min LJ (2009). Attenuation of cuff-induced neointimal formation by overexpression of angiotensin II type 2 receptor-interacting protein 1. Hypertension.

[19] Bundschu K, Knobeloch KP, Ullrich M (2005). Gene disruption of Spred-2 causes dwarfism. J Biol Chem.

[20] Wollert KC, Drexler H (1999). The renin-angiotensin system and experimental heart failure. Cardiovasc Res.

[21] Senbonmatsu T, Ichihara S, Price E, Gaffney FA, Inagami T (2000). Evidence for angiotensin II type 2 receptor-mediated cardiac myocyte enlargement during in vivo pressure overload. J Clin Invest.

[22] Senbonmatsu T, Saito T, Landon EJ (2003). A novel angiotensin II type 2 receptor signaling pathway: possible role in cardiac hypertrophy. EMBO J.

[23] Landon EJ, Inagami T (2005). Beyond the G protein: the saga of the type 2 angiotensin II receptor. Arterioscler Thromb Vasc Biol.

[24] Park TS, Zambidis ET (2009). A role for the renin-angiotensin system in hematopoiesis. Haematologica.

[25] Wuthrich RP (2009). RAS meets SLE. Nephrol Dial Transplant.

[26] Herlitz H, Tarkowski A, Svalander C, Volkmann R, Westberg G (1988). Beneficial effect of captopril on systemic lupus erythematosus-like disease in MRL lpr/lpr mice. Int Arch Allergy Appl Immunol.

[27] Di Benedetto M, Pineau P, Nouet S (2006). Mutation analysis of the 8p22 candidate tumor suppressor gene ATIP/MTUS1 in hepatocellular carcinoma. Mol Cell Endocrinol.

[28] Garcia M, Konoplev S, Morosan C, Abruzzo LV, Bueso-Ramos CE, Medeiros LJ (2007). MALT lymphoma involving the kidney: a report of 10 cases and review of the literature. Am J Clin Pathol.

[29] Smedby KE, Baecklund E, Askling J (2006). Malignant lymphomas in autoimmunity and inflammation: a review of risks, risk factors, and lymphoma characteristics. Cancer Epidemiol Biomarkers Prev.

[30] Isaacson PG, Du MQ (2004). MALT lymphoma: from morphology to molecules. Nat Rev Cancer.

[31] Suzuki Y, Ruiz-Ortega M, Lorenzo O, Ruperez M, Esteban V, Egido J (2003). Inflammation and angiotensin II. Int J Biochem Cell Biol.

[32] Wu L, Iwai M, Li Z (2004). Regulation of inhibitory protein-kappaB and monocyte chemoattractant protein-1 by angiotensin II type 2 receptor-activated Src homology protein tyrosine phosphatase-1 in fetal vascular smooth muscle cells. Mol Endocrinol.

[33] Okamoto T (2006). NF-kappaB and rheumatic diseases. Endocr Metab Immune Disord Drug Targets.

[34] Newton CR, Curran B, Victorino GP (2004). Angiotensin II type 2 receptor effect on microvascular hydraulic permeability. J Surg Res.

[35] Wang ZQ, Moore AF, Ozono R, Siragy HM, Carey RM (1998). Immunolocalization of subtype 2 angiotensin II (AT2) receptor protein in rat heart. Hypertension.

[36] Cao Z, Kelly DJ, Cox A (2000). Angiotensin type 2 receptor is expressed in the adult rat kidney and promotes cellular proliferation and apoptosis. Kidney Int.

[37] Lenkei Z, Palkovits M, Corvol P, Llorens-Cortes C (1996). Distribution of angiotensin II type-2 receptor (AT2) mRNA expression in the adult rat brain. J Comp Neurol.

[38] De Gasparo M, Catt KJ, Inagami T, Wright JW, Unger T (2000). International union of pharmacology. XXIII. The angiotensin II receptors. Pharmacol Rev.

